# Topical cutaneous application of carbon dioxide via a hydrogel for improved fracture repair: results of phase I clinical safety trial

**DOI:** 10.1186/s12891-019-2911-7

**Published:** 2019-11-25

**Authors:** Takahiro Niikura, Takashi Iwakura, Takashi Omori, Sang Yang Lee, Yoshitada Sakai, Toshihiro Akisue, Keisuke Oe, Tomoaki Fukui, Takehiko Matsushita, Tomoyuki Matsumoto, Ryosuke Kuroda

**Affiliations:** 1 Department of Orthopaedic Surgery, Kobe University Graduate School of Medicine, 7-5-1 Kusunoki-cho, Chuo-ku, Kobe, 650-0017 Japan; 2 Department of Orthopaedic Surgery, Hyogo Prefectural Awaji Medical Center, Sumoto, Japan; 3 Division of Biostatistics, Department of Social/Community Medicine and Health Science, Kobe University School of Medicine, Kobe, Japan; 4 Department of Orthopaedic Surgery, Showa University School of Medicine, Tokyo, Japan; 5 Division of Rehabilitation Medicine, Kobe University Graduate School of Medicine, Kobe, Japan; 6 Department of Rehabilitation Science, Kobe University Graduate School of Health Sciences, Kobe, Japan

**Keywords:** Bone, Fracture repair, Carbon dioxide, Blood flow, Clinical trial

## Abstract

**Background:**

Clinicians have very limited options to improve fracture repair. Therefore, it is critical to develop a new clinically available therapeutic option to assist fracture repair biologically. We previously reported that the topical cutaneous application of carbon dioxide (CO_2_) via a CO_2_ absorption-enhancing hydrogel accelerates fracture repair in rats by increasing blood flow and angiogenesis and promoting endochondral ossification. The aim of this study was to assess the safety and efficacy of CO_2_ therapy in patients with fractures.

**Methods:**

Patients with fractures of the femur and tibia were prospectively enrolled into this study with ethical approval and informed consent. The CO_2_ absorption-enhancing hydrogel was applied to the fractured lower limbs of patients, and then 100% CO_2_ was administered daily into a sealed space for 20 min over 4 weeks postoperatively. Safety was assessed based on vital signs, blood parameters, adverse events, and arterial and expired gas analyses. As the efficacy outcome, blood flow at the level of the fracture site and at a site 5 cm from the fracture in the affected limb was measured using a laser Doppler blood flow meter.

**Results:**

Nineteen patients were subjected to complete analysis. No adverse events were observed. Arterial and expired gas analyses revealed no adverse systemic effects including hypercapnia. The mean ratio of blood flow 20 min after CO_2_ therapy compared with the pre-treatment level increased by approximately 2-fold in a time-dependent manner.

**Conclusions:**

The findings of the present study revealed that CO_2_ therapy is safe to apply to human patients and that it can enhance blood flow in the fractured limbs.

**Trial registration:**

This study has been registered in the UMIN Clinical Trials Registry (Registration number: UMIN000013641, Date of registration: July 1, 2014).

## Background

Clinicians have very limited options to biologically improve fracture repair. Although there are a few treatment options such as bone morphogenetic proteins [1–6], low-intensity pulsed ultrasound [7–9], and a pulsed electromagnetic field [10–12], which are used in clinical practice, a search of the existing literature indicated that the effectiveness of these treatments is limited [13–24]. Therefore, it is critical to develop a new clinically available therapeutic option to assist fracture repair biologically.

We previously reported that the topical cutaneous application of carbon dioxide (CO_2_) by means of a CO_2_ absorption-enhancing hydrogel accelerates fracture repair in rats by increasing blood flow and angiogenesis and by promoting endochondral ossification [25]. This CO_2_ therapy induces vasodilation by changing the pH of blood, and it has an immediate effect, increasing the blood flow. In contrast, CO_2_ therapy induces the expression of vascular endothelial growth factor and increases subsequent angiogenesis. It is thought that this therapy increases vascularity via both of these mechanisms. This CO_2_ therapy is thus considered a promising clinically available tool that can be used to assist fracture repair. Therefore, based on the efficacy observed in a pre-clinical study, we conducted a clinical trial involving human subjects. We previously applied the CO_2_ therapy for the treatment of healthy volunteers [26] and found that it caused no adverse events. This study also indicated that CO_2_ therapy induced an artificial Bohr effect in vivo and facilitated the dissociation of oxygen from hemoglobin, leading to local oxygenation in the human body. The present study is the first exploratory trial of CO_2_ therapy involving human patients. The aims of this study were mainly to assess the safety of the technique and to evaluate its efficacy when applied to patients with fractures.

## Methods

### Study design, ethics approval, and informed consent

This study was a prospective, open-label, single-arm, single-center trial. The study protocol was approved by the Institutional Review Board (Approved number: 260008) and the study has been registered in the UMIN Clinical Trials Registry (Registration number: UMIN000013641, Date of registration: July 1, 2014). Prior to the study, we obtained written informed consent from patients who were eligible.

### Inclusion criteria

Patients who fulfilled the following criteria were included in this study: fractures of the lower extremities; either fresh fracture or nonunion; either femur fracture or tibia fracture; within 2 weeks of surgery; aged 15 years and older; provided written informed consent.

### Exclusion criteria

Patients with any of the following were excluded: pathological fractures; dermatologic disease in the fractured limb; active infection in the fractured limb; active bleeding postoperatively; use of any techniques to assist fracture repair such as low-intensity pulsed ultrasound.

### Sample size

We included 20 patients; however, this was not based on any statistical power calculation, as it was difficult to obtain sufficient relevant information to perform the necessary calculations for a preliminary and exploratory study.

### CO_2_ therapy

The CO_2_ absorption-enhancing hydrogel^26^ (NeoChemir, Kobe, Japan) was applied to the skin where we intended to perform trans-cutaneous CO_2_ absorption, that is, the fractured lower extremity of the patients. A polyethylene bag, which can seal the body surface and retain the gas within, was attached to the limb and sealed, and then 100% CO_2_ gas was administered into the bag for 20 min. This treatment was applied to the entire limb, that is, the lower extremity from the hip joint to the toes.

CO_2_ therapy was performed daily for 20 min/day over a 4-week period during hospitalization. We set the treatment period as 4 weeks by considering the duration of hospitalization. The main purpose of this early phase clinical trial was to demonstrate the safety of CO_2_ therapy in human patients for the first time. We considered that therapy safety assessments would be more favorable when performed during hospitalization than in the outpatient clinic. The criteria adopted for the initiation of CO_2_ therapy were no active bleeding, no signs of surgical site infection, and stable general condition after surgery for fresh fractures or nonunion of the lower limb.

### Vital signs

Blood pressure, pulse, body temperature, and SpO_2_ were measured before and after each session of CO_2_ therapy.

### Blood examination

Routine blood examination was performed before and after surgery. Clinically significant values were checked by physicians to diagnose any possible systemic side effects of the CO_2_ therapy.

### Arterial gas analysis

Arterial gas analysis was performed immediately before and after CO_2_ therapy on day 14 after the initiation of treatment. Arterial blood was collected from the femoral artery.

### Expired gas analysis

Expired gas analysis was performed before and during CO_2_ therapy on day 14 after the initiation of treatment using a Cpex-1 ventilatory expired gas analysis system (NIHON MEDIX CO., LTD., Chiba, Japan).

### Adverse events

Physicians monitored the patients daily for any adverse events including systemic and local events during the 4-week treatment period and at each outpatient clinic visit following discharge from the hospital.

### Measurement of blood flow in the patients’ limbs

Blood flow in the patients’ limbs, both in the fractured limb and the contra–lateral healthy limb, was measured using a laser Doppler blood flow meter (Cyber Med CDF2000; Nexis, Fukuoka, Japan). Blood flow was also measured at the level of the fracture site and at a point 5 cm from the fracture site in both limbs. Blood flow was measured continuously from before the commencement of CO_2_ therapy to 20 min after the 20-min period of CO_2_ therapy. These blood flow measurements were obtained on three separate days, specifically the first day of CO_2_ therapy and on days 14 and 28 after the initiation of CO_2_ therapy.

### Follow-up

After discharge from the hospital, the patients were followed-up routinely in an outpatient clinic. The follow-up period was defined as the time from the first day of CO_2_ therapy to the most recent outpatient visit.

### Radiographic and clinical fracture union assessment

Radiographic and clinical fracture unions were assessed during the routine follow-ups in the outpatient clinic after discharge from the hospital. Completion of bony bridging at three of the four cortices for diaphyseal fractures and disappearance of the fracture line for epiphyseal and metaphyseal fractures were judged as radiographic fracture union. Clinical fracture union was assumed when a patient was able to bear full weight on the affected limb without pain.

### Statistics

Each patient was assigned an identification number, and all information was maintained confidential. The investigator filled out the data for each patient in a case report form, which was transferred to a data manager. The dataset compiled after data cleaning by the data manager was transferred to a biostatistician who performed the appropriate statistical analyses.

The patients’ baseline characteristics were summarized as summary statistics (number of patients, mean, standard deviation, minimum, median, and maximum) for continuous valuables and as categorical frequency and proportion for nominal variables.

The outcomes of the arterial gas and expired gas analyses were obtained on day 14 of CO_2_ therapy. For each outcome, the mean values with the respective 95% confidence intervals were determined for the differences between pre-treatment and at 20 min after the initiation of treatment. As the endpoint of blood flow, we estimated the blood flow ratio for each patient defined as the ratio of blood flow at 20 min after treatment relative to that at pre-treatment. The mean, range, and 95% confidence interval of the blood flow ratio were calculated for the endpoint, for both the measurement sites and for each of the three measurement days (days 1, 14, and 28). We calculated *p*-values for the endpoint using a Wilcoxon signed rank test with a null hypothesis that the population mean of the blood flow ratio was 1. A small *p*-value supports the rejection of the aforementioned null hypothesis. We adopted a significance level of 0.05 and did not consider adjusting any multiplicity for the statistical test because this study constituted an exploratory examination. The additional data points were as follows: pre-treatment, 5 min after the initiation of the CO_2_ therapy, 10 min after the initiation of the CO_2_ therapy, 15 min after the initiation of the CO_2_ therapy, 20 min after the initiation of the CO_2_ therapy, 5 min after the termination of the CO_2_ therapy, 10 min after the termination of the CO_2_ therapy, 15 min after the termination of the CO_2_ therapy, and 20 min after the termination of the CO_2_ therapy. We also calculated the blood flow ratio for each patient defined as the ratio of blood flow at each data point relative to that at pre-treatment.

Additionally, we conducted sub-group analyses to investigate the effects of age, type of osteosynthesis, time of initiation of weight bearing, affected bone (femur or tibia), and smoking on the blood flow-enhancing effects of CO_2_ therapy. We calculated *p*-values using the Mann–Whitney U test to compare two groups and the Kruskal–Wallis test to compare three groups. All statistical analyses were performed using SAS software version 9.4 (SAS Institute, Cary, NC).

## Results

### Patients’ baseline characteristics

Twenty patients were enrolled to the trial in accordance with the study design. The patients’ baseline characteristics are summarized in Table 1. One of the patients (ID 11003) dropped out from the study on the third day after the initiation of CO_2_ therapy. This was because the patient expressed a wish to receive low-intensity pulsed ultrasound for fracture treatment. Therefore, all analyses were conducted using the data obtained from the remaining 19 patients. Of the 19 patients for whom complete data analyses were performed, 13 were men and six were women, with a mean age of 48.7 years (range, 23–76 years) and mean body mass index of 25.6 (range, 18.8–30.1). Among the fractures treated, seven were fresh fractures and 12 were nonunions. The fractured bone was the femur in eight patients and tibia in 11 patients. The percentage of smokers among the 19 patients was 73.7% (12 current smokers and two previous smokers). The mean number of days from surgery to the initiation of CO_2_ therapy was 7.9 (range, 2–12), and the mean follow-up period was 27.5 months (range, 9–48).

**Table 1:** Patients’ baseline characteristics

Patient ID	Age range	Sex	BMI	Fresh fracture or Nonunion	Affected bone	Fracture level	Smoking	Comorbidities	Days from surgery to CO_2_ therapy	Follow-up (months)
11,001	50–59	M	28.7	Nonunion	Femur	32	Current	Previous infection	9	48
11,002	60–69	F	22.4	Nonunion	Femur	31		RA, AFF	10	48
11,003	40–49	M	25.7	Fresh fracture	Tibia	41	Current		11	26
11,004	40–49	M	30.1	Fresh fracture	Tibia	41	Current	Gustilo type II open fracture	10	24
11,005	60–69	F	23.1	Fresh fracture	Tibia	41		RA, Adult Still’s disease, HT, HL	11	9
11,006	20–29	M	25.5	Nonunion	Tibia	43	Current		7	33
11,007	40–49	F	18.8	Nonunion	Femur	32 + 33			6	46
11,008	20–29	M	27.3	Nonunion	Femur	32	Current		7	17
11,009	70–79	F	29.4	Fresh fracture	Tibia	33		Graves’ disease, HT	12	15
11,010	20–29	M	23.6	Nonunion	Tibia	42	Current		9	31
11,011	40–49	M	27.4	Fresh fracture	Tibia	41	Current		7	24
11,012	40–49	M	23.8	Nonunion	Tibia	43	Current		9	42
11,013	30–39	M	24.9	Fresh fracture	Tibia	41	Previous		9	36
11,014	60–69	M	24.8	Fresh fracture	Femur	32	Previous	DM, HT	12	14
11,015	40–49	F	25.3	Fresh fracture	Tibia	44	Current	Uterus myoma, Ovarian tumor	6	12
11,016	40–49	M	22	Nonunion	Tibia	42	Current		9	36
11,017	50–59	M	27.9	Nonunion	Femur	32	Current	Duodenum ulcer, Depression	6	15
11,018	60–69	F	25	Nonunion	Tibia	32	Current	RA, HT	7	24
11,019	50–59	M	29.2	Nonunion	Femur	31			2	24
11,020	50–59	M	26.4	Nonunion	Femur	32	Current		2	25

*M* male, *F* female, *BMI* body mass index, *RA* rheumatoid arthritis, *AFF* atypical femoral fracture, *HT* hypertension, *HL* hyperlipidemia, *DM* diabetes mellitus

Fracture level was coded with the AO/OTA classification. AO: Arbeitsgemeinschaft für Osteosynthesefragen, OTA: Orthopaedic Trauma Association

Smoking: A current smoker is a patient who smoked at the time of initiation of the treatment at the author’s institute. They were advised to quit smoking in order to be treated at the author’s institute. Previous smoker means a patient who quit smoking at least 1 year prior to the initiation of the treatment at the author’s institute

Days from surgery to CO_2_ therapy: Days from surgery to initiation of the CO_2_ therapy

Follow-up (months): Months from initiation of the CO_2_ therapy to the most recent outpatient clinic visit

### Vital signs

**Fig. 1:**
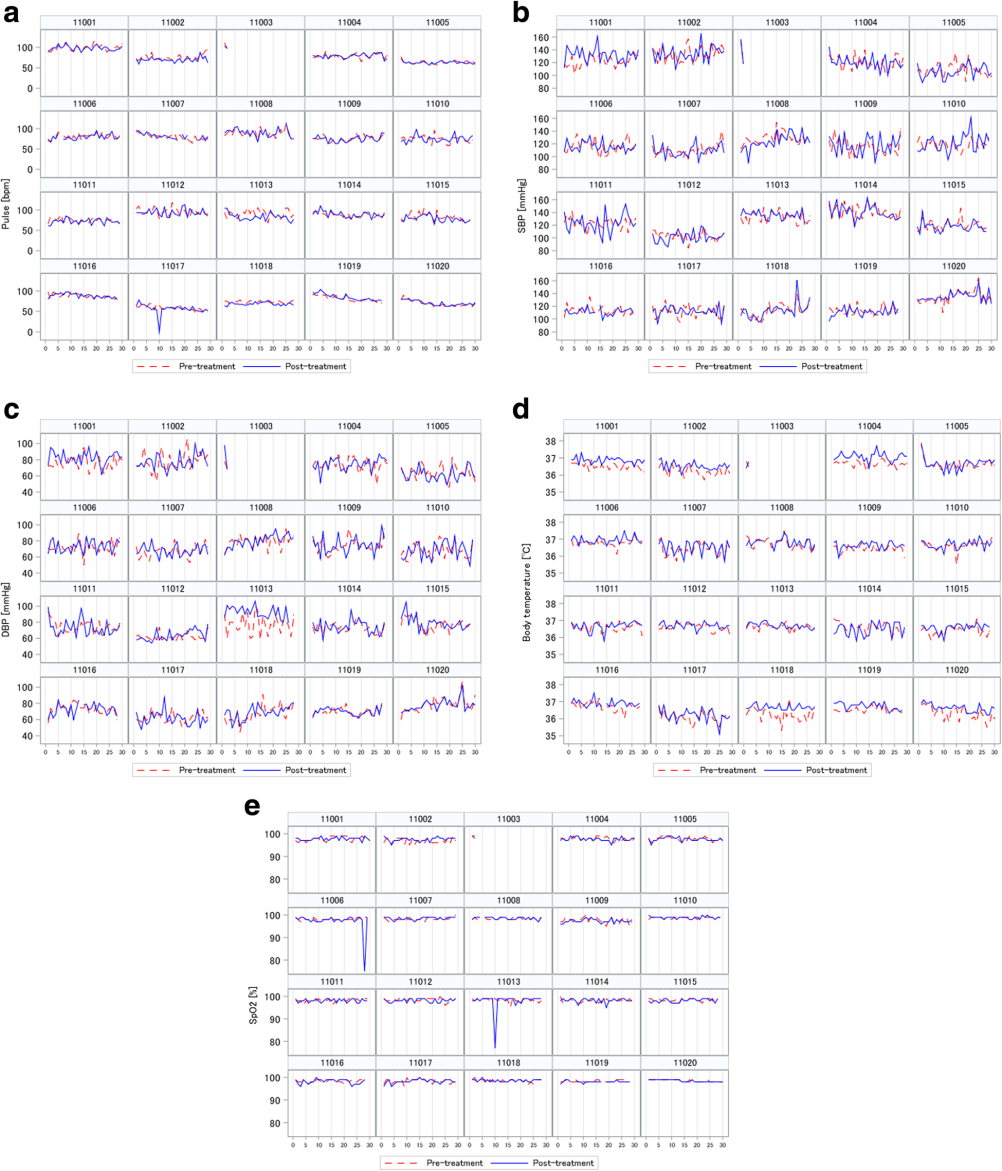
Vital signs of patient cohort. The horizontal axis represents days. The dashed line shows the values measured before CO_2_ therapy, and the solid line shows the values measured after CO_2_ therapy. **a** Pulse, **b** systolic blood pressure, **c** diastolic blood pressure, **d** body temperature, and **e** SpO_2_

Figure 1 shows the vital signs before and after CO_2_ therapy for 28 consecutive days. There were no marked changes in vital signs before and after CO_2_ therapy.

### Blood examination

There were no marked deviations from the standard postoperative course with respect to blood examination (data not shown); there were no liver or kidney function disorders.

### Arterial gas analysis

Arterial gas analysis revealed that there were no significant differences in each parameter before and after CO_2_ therapy (Table 2). Notably, no hypercapnia was observed.

**Table 2:** Arterial gas analysis

	Unit	Before the CO_2_ therapy	After the CO_2_ therapy	Difference between the means	95%CI for the difference
BE	mmol/L	−0.05 (− 1.60 to 2.10)	0.18 (−2.30 to 2.50)	0.23 (−1.90 to 1.40)	[− 0.11 to 0.57]
HCO_3_-	mmol/L	24.18 (22.90–26.50)	24.53 (22.20–27.40)	0.35 (−1.40 to 1.50)	[−0.02 to 0.71]
O_2_SAT	%	97.50 (96.10–99.90)	97.37 (92.40–100.00)	−0.13 (− 4.00 to 3.30)	[− 0.90 to 0.64]
PaCO_2_	mmHg	40.13 (33.70–43.80)	41.02 (34.30–47.50)	0.89 (−6.00 to 7.50)	[−0.50 to 2.28]
PaO_2_	mmHg	93.54 (76.80–132.00)	99.14 (76.40–167.00)	5.60 (−13.00 to 86.90)	[−5.36 to 16.56]
pH	mU/dL	7.40 (7.36–7.45)	7.39 (7.35–7.44)	−0.00 (−0.06 to 0.04)	[−0.02 to 0.01]
Deoxy-Hb	%	2.44 (0.10–3.80)	2.58 (0.00–7.60)	0.14 (−3.20 to 4.10)	[−0.62 to 0.90]
Oxy-Hb	%	95.21 (92.60–97.10)	95.24 (92.20–97.60)	0.04 (−2.40 to 3.20)	[−0.61 to 0.68]

Values are expressed as mean (range). *n* = 19

*BE* base excess, *HCO*_*3*_*-* bicarbonate ion, *O*_*2*_*SAT* oxygen saturation, *PaCO*_*2*_ partial pressure of carbon dioxide in arterial blood, *PaO*_*2*_ partial pressure of oxygen in arterial blood, *pH* power of hydrogen, *Deoxy-Hb* deoxyhemoglobin, *Oxy-Hb* oxygenated hemoglobin, *95%CI* 95% confidence interval

### Expired gas analysis

There were no significant differences in each parameter before and during CO_2_ therapy (Table 3).

**Table 3:** Expired gas analysis data

	Unit	Before CO_2_ therapy	During CO_2_ therapy	Difference between the means	95%CI for the difference
ETCO_2_	%	4.78 (4.16–5.59)	4.76 (4.28–5.51)	−0.02 (− 0.34 to 0.35)	[−0.13 to 0.09]
ETO_2_	%	16.05 (4.57–17.43)	16.08 (14.08–17.09)	0.03 (−0.96 to 0.95)	[−0.21 to 0.28]
R	N/A	0.98 (0.83–1.25)	1.00 (0.79–1.23)	0.02 (−0.06 to 0.21)	[−0.02 to 0.06]
VCO_2_	mL/min	279.58 (136.36–570.26)	280.05 (54.75–575.90)	0.47 (− 116.0 to 93.08)	[−22.66 to 23.61]
VE	L/min	9.48 (5.99–15.30)	9.47 (2.20–15.20)	−0.01 (−4.54 to 3.30)	[− 0.85 to 0.82]
VE/VCO_2_	N/A	38.74 (20.54–59.23)	39.71 (23.26–61.19)	0.97 (−7.48 to 12.58)	[−1.38 to 3.32]
VE/VO_2_	N/A	36.82 (20.39–49.54)	38.20 (26.04–49.56)	1.39 (−8.02 to 12.88)	[−1.51 to 4.29]
VO_2_	mL/min	274.04 (63.67–499.26)	272.64 (53.00–518.14)	−1.40 (− 105.4 to 144.33)	[−32.32 to 29.52]

Values are expressed as mean (range). *n* = 19

*ETCO*_*2*_ end tidal carbon dioxide, *ETO*_*2*_ end tidal oxygen, *R* respiratory exchange ratio (VCO_2_/VO_2_), *VCO*_*2*_ carbon dioxide output volume, *VE* expiratory minute ventilation, *VO*_*2*_ oxygen uptake volume, *95%CI* 95% confidence interval, *N/A* not applicable

### Adverse events

No systemic or local adverse events were observed in any of the patients, and no skin-related reactions were observed due to application of the hydrogel to the skin. Moreover, there were no surgical site infections or clinical hypercapnia.

### Radiographic and clinical fracture union assessment

**Fig. 2:**
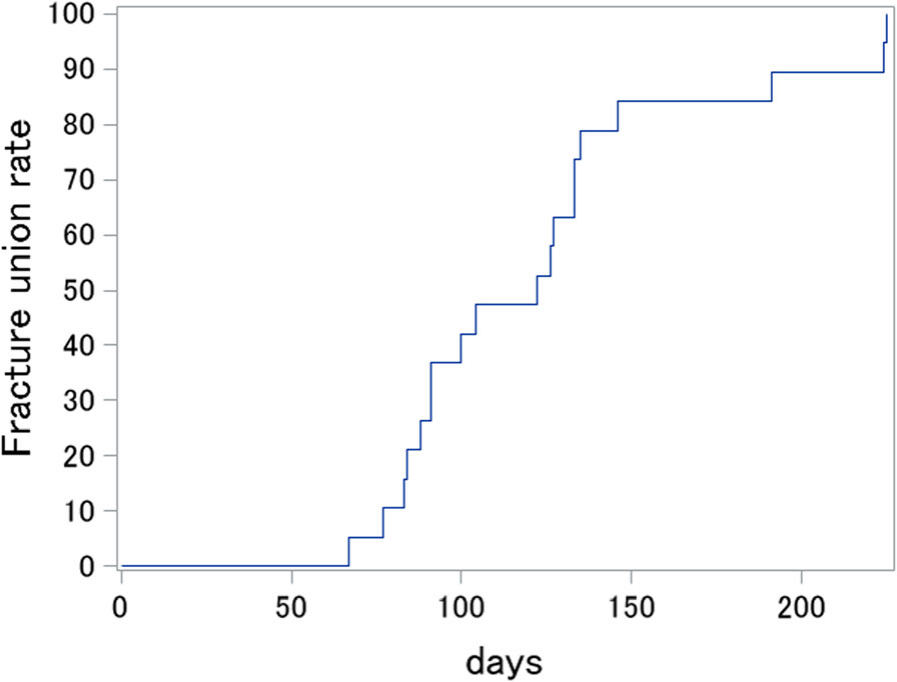
Kaplan–Meier survival curve of fracture union. The horizontal axis represents the days from surgery to treat fracture/nonunion. The vertical axis represents the radiographic fracture union rate

Radiographic fracture union was completed for all patients (Fig. 2). Clinical fracture union was also achieved for all patients.

### Measurement of blood flow in patients’ limbs

**Fig. 3:**
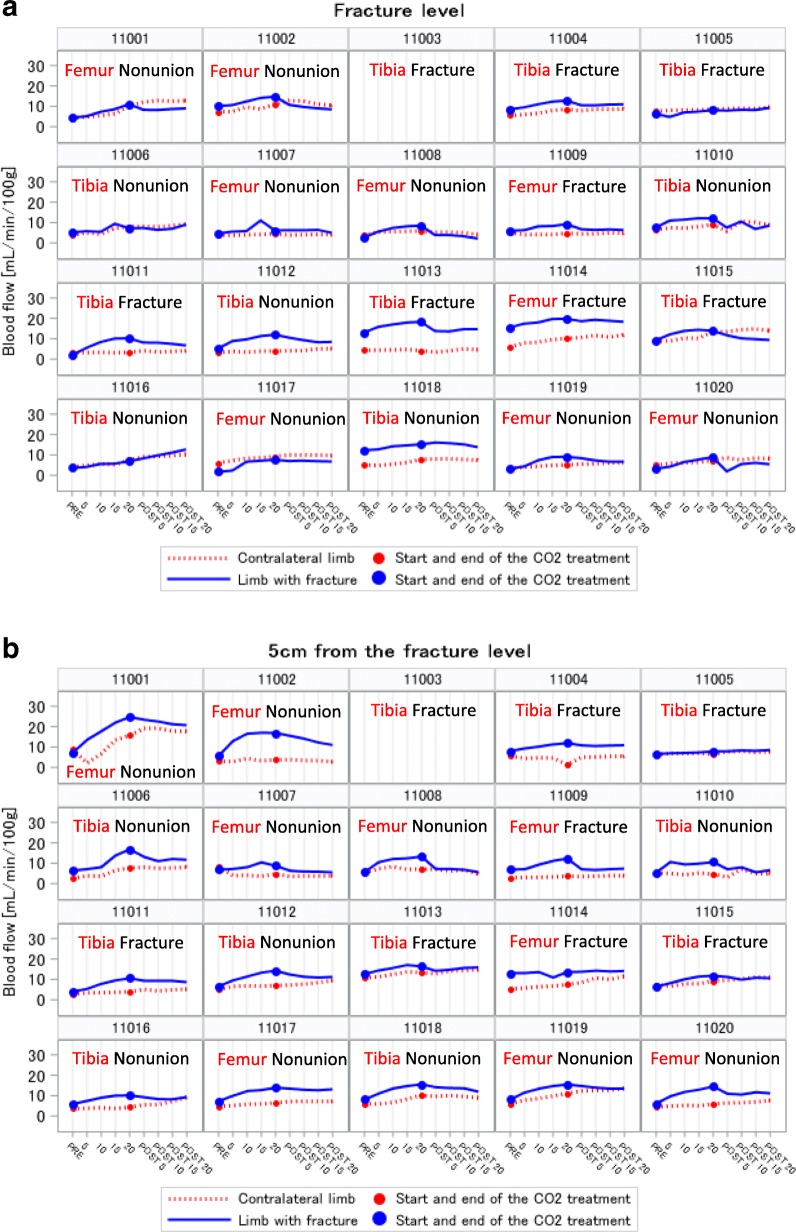
Blood flow in patient limbs on day 28 after the initiation of CO_2_ therapy. The horizontal axis indicates the time course. Blood flow was measured continuously from prior to the commencement of the CO_2_ therapy (PRE) to 20 min after (POST) a 20-min session of the CO_2_ therapy. The vertical axis shows the blood flow. The affected bone (femur or tibia) and whether the fracture is a fresh fracture or a fracture with nonunion are indicated. The solid line shows the blood flow in the fractured limb and the dotted line shows the blood flow in the contra–lateral healthy limb. Filled circles on the lines indicate the time point of the start and end of the CO_2_ therapy. **a** Blood follow measured at the fracture level. **b** Blood follow measured at a point 5 cm from the fracture level

Figure 3 illustrates the blood flow over time for each patient on day 28 at the fracture level (Fig. 3a) and at a site 5 cm from the fracture level (Fig. 3b). The solid and dotted lines represent the values for the fractured and contra–lateral healthy limb, respectively. Blood flow at 20 min in the fractured limb was higher than that in the contra–lateral healthy limb in 16 of the 19 patients (84.2%) at the fracture level and in all 19 patients (100%) at the site 5 cm from the fracture level. No marked differences in the dynamic tendency of blood flow were observed between patients with femur and tibia fractures or between those with fresh fractures and nonunions.

Table 4 summarizes the endpoint and the ratio of blood flow in the patients’ fractured limbs from pre-treatment to post-treatment on days 1, 14, and 28. Based on these data, it was evident that CO_2_ therapy promoted an increase in blood flow in the fractured limbs. The mean values increased in a time-dependent manner for both fracture and adjacent sites, and the mean ratios showed an approximate 2-fold increase on day 28. The small *p*-values in both the tables indicate an increase in blood flow attributable to CO_2_ therapy compared to pre-treatment measurements.

**Table 4:** Increase in blood flow promoted by the CO_2_ therapy in the fractured limb of patients

Measuring site	Treatment day	Mean (range) [95%CI]	*p*-value
Fracture level	1	1.414 (0.970–2.846) [1.218–1.611]	*p* < 0.00001
	14	1.764 (1.156–3.152) [1.491–2.036]	*p* < 0.00001
	28	2.137 (1.236–5.100) [1.602–2.673]	p < 0.00001
5 cm from the fracture level	1	1.478 (1.010–2.000) [1.344–1.612]	p < 0.00001
	14	1.855 (1.168–2.660) [1.623–2.087]	p < 0.00001
	28	1.997 (1.038–3.431) [1.694–2.300]	p < 0.00001

Blood flow increase is demonstrated by the ratio of blood flow measured after 20 min of CO_2_ therapy to that at pre-treatment. Number of patients = 19. *p*-value: calculated using Wilcoxon signed rank test with a null hypothesis that the population mean of the blood flow ratio is 1

We then performed a sub-group analysis of enhanced blood flow by dividing patients into groups of ≤45 (*n* = 9) and > 45 (*n* = 10) years of age, and the results are presented in Table 5. We found a statistically significant difference on day 1, measured at the fracture level (*p* = 0.030). The increase in blood flow was higher in the aged group. However, we did not find significant differences for the other conditions.

**Table 5:** Sub-group analysis regarding the influence of age on the increase in blood flow by the CO_2_ therapy in the fractured limb of patients

Measuring site	Treatment day	Age	n	Mean (range) [95%CI]	*p*-value (1)	*p*-value (2)
Fracture level	1	≤45	9	1.221 (0.970–1.473) [1.106–1.336]	0.008	0.030
		> 45	10	1.589 (1.146–2.846) [1.238–1.940]	0.002	
	14	≤45	9	1.750 (1.184–3.152) [1.289–2.211]	0.004	0.97
		> 45	10	1.777 (1.156–2.829) [1.372–2.181]	0.002	
	28	≤45	9	2.101 (1.326–5.100) [1.135–3.068]	0.004	0.97
		> 45	10	2.170 (1.236–4.471) [1.432–2.907]	0.002	
5 cm from the fracture level	1	≤45	9	1.453 (1.010–1.705) [1.290–1.615]	0.004	0.77
		> 45	10	1.501 (1.060–2.000) [1.260–1.742]	0.002	
	14	≤45	9	1.811 (1.314–2.440) [1.506–2.116]	0.004	0.78
		> 45	10	1.895 (1.168–2.660) [1.490–2.300]	0.002	
	28	≤45	9	1.908 (1.279–2.625) [1.507–2.309]	0.004	0.71
		> 45	10	2.077 (1.038–3.431) [1.555–2.600]	0.002	

Blood flow increase is demonstrated by the ratio of blood flow measured after 20 min of CO_2_ therapy to that at pre-treatment. n: number of patients. *p*-value (1): calculated using Wilcoxon signed rank test with a null hypothesis that the population mean of the blood flow ratio is 1. *p*-value (2): calculated using Mann–Whitney U test to compare the two groups (Age ≤ 45 versus > 45)

We also performed a sub-group analysis of enhanced blood flow by dividing patients based on treatment with intramedullary (IM) nailing (*n* = 9), plate (*n* = 9), and neither IM nailing nor plate (*n* = 1), and the results are presented in Table 6. We found a statistically significant difference on day 14, measured at the fracture level (*p* = 0.040). The increase in blood flow was higher in the IM nailing group. However, we did not find significant differences for the other conditions.

**Table 6:** Sub-group analysis regarding the influence of the type of osteosynthesis on the increase in blood flow by the CO_2_ therapy in the fractured limb of patients

Measuring site	Treatment day	Type of osteosynthesis	n	Mean (range) [95%CI]	*p*-value (1)	*p*-value (2)
Fracture level	1	IMN	9	1.523 (0.970–2.846) [1.096–1.950]	0.008	0.41
		Plate	9	1.336 (1.080–1.618) [1.192–1.480]	0.004	
		Other	1	1.146		
	14	IMN	9	2.124 (1.158–3.152) [1.642–2.606]	0.004	0.040
		Plate	9	1.448 (1.156–1.720) [1.278–1.618]	0.002	
		Other	1	1.362		
	28	IMN	9	2.363 (1.236–4.471) [1.533–3.192]	0.004	0.50
		Plate	9	1.888 (1.306–5.100) [0.952–2.824]	0.004	
		Other	1	2.353		
5 cm from the fracture level	1	IMN	9	1.451 (1.010–2.000) [1.175–1.726]	0.004	0.36
		Plate	9	1.468 (1.164–1.705) [1.334–1.602]	0.004	
		Other	1	1.816		
	14	IMN	9	2.119 (1.168–2.660) [1.701–2.538]	0.004	0.09
		Plate	9	1.592 (1.206–2.091) [1.390–1.793]	0.004	
		Other	1	1.851		
	28	IMN	9	2.216 (1.038–3.431) [1.680–2.752]	0.004	0.20
		Plate	9	1.759 (1.215–2.625) [1.351–2.167]	0.004	
		Other	1	2.167		

Blood flow increase is demonstrated by the ratio of blood flow measured after 20 min of CO_2_ therapy to that at pre-treatment. n: number of patients. *p*-value (1): calculated using Wilcoxon signed rank test with a null hypothesis that the population mean of the blood flow ratio is 1. *p*-value (2): calculated using Kruskal–Wallis test to compare the three groups (IMN versus plate versus other). IMN: intramedullary nailing

Next, we performed a sub-group analysis of enhanced blood flow by dividing patients based on the initiation of weight bearing, specifically ≤5 weeks (*n* = 7) and > 5 weeks (*n* = 12) post-operation, and the results are presented in Table 7. We found a statistically significant difference on day 28, measured 5 cm from the fracture level (*p* = 0.047). The increase in blood flow was higher in the group with earlier initiation of weight bearing. However, we did not find significant differences for the other conditions.

**Table 7:** Sub-group analysis regarding the influence of weight bearing on the increase in blood flow by the CO_2_ therapy in the fractured limb of patients

Measuring site	Treatment day	Weight bearing (weeks)	n	Mean (range) [95%CI]	*p*-value (1)	*p*-value (2)
Fracture level	1	≤5	7	1.490 (1.080–2.846) [0.922–2.057]	0.016	0.77
		> 5	12	1.371 (0.970–1.825) [1.215–1.526]	< 0.001	
	14	≤5	7	2.119 (1.158–3.152) [1.439–2.800]	0.016	0.14
		> 5	12	1.556 (1.156–2.145) [1.355–1.758]	< 0.001	
	28	≤5	7	2.190 (1.277–3.148) [1.455–2.925]	0.016	0.64
		> 5	12	2.107 (1.236–5.100) [1.284–2.929]	< 0.001	
5 cm from the fracture level	1	≤5	7	1.449 (1.060–2.000) [1.177–1.721]	0.016	0.74
		> 5	12	1.495 (1.010–2.000) [1.317–1.673]	< 0.001	
	14	≤5	7	2.116 (1.168–2.660) [1.544–2.688]	0.016	0.19
		> 5	12	1.703 (1.206–2.346) [1.500–1.906]	< 0.001	
	28	≤5	7	2.397 (1.038–3.431) [1.682–3.112]	0.016	0.047
		> 5	12	1.764 (1.215–2.585) [1.510–2.018]	< 0.001	

Blood flow increase is demonstrated by the ratio of blood flow measured after 20 min of CO_2_ therapy to that at pre-treatment. n: number of patients. *p*-value (1): calculated using Wilcoxon signed rank test with a null hypothesis that the population mean of the blood flow ratio is 1. *p*-value (2): calculated using Mann–Whitney U test to compare the two groups (weight bearing initiated ≤5 weeks versus > 5 weeks)

We then performed a sub-group analysis of enhanced blood flow by dividing patients based on the affected bones, namely the femur (*n* = 8) and tibia (*n* = 11), and the results are presented in Table 8. We found a statistically significant difference on day 14, measured at the fracture level (*p* = 0.015). The increase in blood flow was higher in the femur group. However, we did not find significant differences for the other conditions.

**Table 8:** Sub-group analysis regarding the influence of affected bone on the increase in blood flow by the CO_2_ therapy in the fractured limb of patients

Measuring site	Treatment day	Affected bone	n	Mean (range) [95%CI]	*p*-value (1)	*p*-value (2)
Fracture level	1	Femur	8	1.518 (1.153–2.846) [1.054–1.982]	0.008	0.59
		Tibia	11	1.339 (0.970–1.825) [1.164–1.514]	0.002	
	14	Femur	8	2.162 (1.158–3.152) [1.637–2.687]	0.008	0.015
		Tibia	11	1.474 (1.156–2.145) [1.283–1.665]	< 0.001	
	28	Femur	8	2.468 (1.277–4.471) [1.548–3.387]	0.008	0.30
		Tibia	11	1.897 (1.236–5.100) [1.153–2.641]	< 0.001	
5 cm from the fracture level	1	Femur	8	1.428 (1.060–2.000) [1.198–1.659]	0.008	0.28
		Tibia	11	1.514 (1.010–2.000) [1.321–1.707]	< 0.001	
	14	Femur	8	2.073 (1.168–2.660) [1.578–2.567]	0.008	0.20
		Tibia	11	1.697 (1.206–2.346) [1.478–1.917]	< 0.001	
	28	Femur	8	2.167 (1.038–3.431) [1.489–2.845]	0.008	0.48
		Tibia	11	1.874 (1.215–2.625) [1.565–2.182]	< 0.001	

Blood flow increase is demonstrated by the ratio of blood flow measured after 20 min of CO_2_ therapy to that at pre-treatment. n: number of patients. *p*-value (1): calculated using Wilcoxon signed rank test with a null hypothesis that the population mean of the blood flow ratio is 1. *p*-value (2): calculated using Mann–Whitney U test to compare the two groups (femur versus tibia)

Finally, we performed a sub-group analysis of enhanced blood flow by dividing patients into non-smoker (*n* = 5), current smoker (*n* = 12), and previous smoker (*n* = 2) groups, and the results are presented in Table 9. We found statistically significant differences on days 14 and 28, measured at 5 cm from the fracture level (*p* = 0.036 each). However, we did not find significant differences for the other conditions.

**Table 9:** Sub-group analysis regarding the influence of smoking on the increase in blood flow by the CO_2_ therapy in the fractured limb of patients

Measuring site	Treatment day	Smoking status	n	Mean (range) [95%CI]	*p*-value (1)	*p*-value (2)
Fracture level	1	Non-smoker	5	1.377 (1.153–1.618) [1.117–1.637]	0.063	0.65
		Current smoker	12	1.429 (0.970–2.846) [1.109–1.749]	< 0.001	
		Previous smoker	2	1.420 (1.366–1.473) [0.739–2.100]	0.50	
	14	Non-smoker	5	1.656 (1.156–2.057) [1.202–2.110]	0.063	0.055
		Current smoker	12	1.899 (1.184–3.152) [1.500–2.299]	< 0.001	
		Previous smoker	2	1.220 (1.158–1.281) [0.436–2.003]	0.50	
	28	Non-smoker	5	1.692 (1.306–2.844) [0.884–2.500]	0.063	0.12
		Current smoker	12	2.455 (1.236–5.100) [1.664–3.247]	< 0.001	
		Previous smoker	2	1.343 (1.277–1.408) [0.515–2.170]	0.50	
5 cm from the fracture level	1	Non-smoker	5	1.457 (1.164–2.000) [1.062–1.852]	0.063	0.93
		Current smoker	12	1.503 (1.010–2.000) [1.337–1.669]	< 0.001	
		Previous smoker	2	1.382 (1.060–1.705) [−2.71–5.478]	0.50	
	14	Non-smoker	5	1.719 (1.206–2.611) [1.064–2.374]	0.063	0.036
		Current smoker	12	2.015 (1.453–2.660) [1.751–2.278]	< 0.001	
		Previous smoker	2	1.241 (1.168–1.314) [0.313–2.168]	0.50	
	28	Non-smoker	5	1.842 (1.215–3.018) [0.942–2.742]	0.063	0.036
		Current smoker	12	2.200 (1.488–3.431) [1.868–2.531]	< 0.001	
		Previous smoker	2	1.170 (1.038–1.302) [−.504–2.843]	0.50	

Blood flow increase is demonstrated by the ratio of blood flow measured after 20 min of CO_2_ therapy to that at pre-treatment. n: number of patients. *p*-value (1): calculated using Wilcoxon signed rank test with a null hypothesis that the population mean of the blood flow ratio is 1. *p*-value (2): calculated using Kruskal–Wallis test to compare the three groups (Non-smoker versus current smoker versus previous smoker)

Blood flow data in the contra–lateral healthy leg were also analyzed. We found a statistically significant increase in blood flow in the contra–lateral healthy leg, and the results are presented in Table 10.

**Table 10:** Increase in blood flow promoted by the CO_2_ therapy in the contra–lateral non-fractured limb of patients

Measuring site	Treatment day	Mean (range) [95%CI]	*p*-value
Fracture level	1	1.220 (0.875–1.533) [1.140–1.300]	< 0.0001
	14	1.431 (0.831–2.139) [1.262–1.600]	< 0.0001
	28	1.493 (0.941–2.429) [1.311–1.675]	< 0.0001
5 cm from the fracture level	1	1.204 (0.438–1.611) [1.068–1.339]	0.009
	14	1.396 (0.669–2.079) [1.228–1.564]	< 0.001
	28	1.335 (0.236–2.962) [1.071–1.599]	0.011

Blood flow increase is demonstrated by the ratio of blood flow measured after 20 min of CO_2_ therapy to that at pre-treatment. Number of patients = 19. *p*-value: calculated using Wilcoxon signed rank test with a null hypothesis that the population mean of the blood flow ratio is 1

## Discussion

Given that CO_2_ therapy introduces CO_2_ into the body, there have been concerns regarding the potential occurrence of hypercapnia. In this study, however, we demonstrated that the CO_2_ therapy that we used causes no adverse events including hypercapnia in patients. The successful verification of the safety of CO_2_ therapy was the main outcome of the current clinical trial. This favorable outcome supports the validity of continuing assessments of this CO_2_ therapy in further clinical trials with patients.

In addition to the effect of accelerating fracture healing, various positive effects of CO_2_ therapy have been reported in pre-clinical studies. One example is the effects of CO_2_ therapy on muscles, which include muscle fiber switching in skeletal muscle [27], acceleration of muscle injury repair [28], and acceleration of the performance of endurance exercise [29]. Another example relates to the effects of this therapy on tumors. CO_2_ therapy has been demonstrated to have inherent antitumor effects [30–33] by suppressing metastasis [33, 34], enhancing the antitumor effect of radiation therapy [35, 36], and suppressing bone destruction caused by bone metastasis [37]. All of these are targets that warrant further examination in clinical trials. The clinical trial reported herein is the first such trial involving human patients, and therefore, the information we provide regarding the proven safety of CO_2_ therapy will be valuable to other investigators conducting future clinical trials for various diseases.

We focused on blood flow in this study because it is one of the most critical factors associated with fracture repair. Poor vascularity adversely affects fracture repair [38–40] and is a risk factor for nonunion [41]; it has also been reported as a target for treatment to improve nonunion [42]. Angiogenesis is a key component of bone repair [43, 44] and modern fracture fixation techniques such as biological osteosynthesis and minimally invasive plate osteosynthesis, which are aimed at preserving vascularity around the fracture site to enhance fracture healing [45–49]. Therefore, we adopted blood flow in the fractured limb as a surrogate endpoint signifying a positive effect on fracture repair.

Based on the measurements obtained in the present study, it is evident that CO_2_ therapy can effectively increase blood flow in the fractured limbs. Additionally, in the majority of patients, we recorded higher blood flow in the fractured limb than in the contra–lateral healthy limb (Fig. 3). As indicated in Table 4, blood flow showed a time-dependent increase throughout treatment. This phenomenon can be attributed to one or both of the following processes. First, the effect of increased blood flow promoted by CO_2_ therapy is reinforced by the continuation of daily CO_2_ therapy. Second, the vascularity of the fractured limb itself increases with time after surgery, which reflects the course of the healing process. It is possible that new blood vessel formation occurs with time after surgery. Moreover, there is an increase in the number of blood vessels that can respond to the effect of increased blood flow promoted by CO_2_ therapy. We additionally analyzed the data of blood flow in the contra–lateral healthy leg as shown in Table 10. We also found a statistically significant increase in blood flow in the contra–lateral healthy leg. This could be because the CO_2_ therapy induced some systemic effects to increase blood flow, and the increased blood flow observed in the fractured limbs was not induced only by the fracture healing process.

In the present study, we measured blood flow at two different points in the fractured limb, specifically at the fracture level and at a site 5 cm from the fracture level. In some cases, during surgery, the skin at the fracture site is incised, and this raises concerns because surgical incision might disrupt the vascularity of soft tissue at the fracture level. Therefore, in the present study, we decided to additionally measure blood flow at a point in the ipsilateral limb slightly removed from the fracture level. Consequently, it was evident that an increase in blood flow was promoted at both the fracture level and its surroundings. The increase in blood flow in the fractured limb was accordingly deemed to contribute to fracture repair. A similar tendency of increased blood flow was observed for cases of femur and tibia fractures, and in both fresh fractures and fractures with nonunion. However, further in-depth analysis is needed to determine the possible differences between femur and tibia fractures and fresh fractures and those with nonunion.

Despite the small number of patients, owing to the nature of this small-sized, early phase clinical trial, we performed sub-group analyses. We found some statistically significant differences; however, we cannot definitely conclude that the factors analyzed affect the blood flow-enhancing effects of the CO_2_ therapy. It is still unclear whether age, type of osteosynthesis, time of initiation of weight bearing, affected bone (femur or tibia), and smoking status affect CO_2_ therapy outcomes to enhance blood flow in the present small-sized clinical trial. Although it cannot be neglected that the number of patients was small in the present study, we found some significant findings. It seems that age does not significantly contribute to the effect of CO_2_ therapy on enhance blood flow. It is possible that IM nailing affects the bone circulation by damaging the endosteal blood supply; in contrast, IM nailing can preserve the periosteal blood supply. Therefore, it is possible that CO_2_ therapy is more effective in enhancing blood flow around the bone in the fractured limb treated by IM nailing. It is possible that an earlier weight bearing reinforces the effect of the CO_2_ therapy to enhance blood flow. Further, the effect of the CO_2_ therapy to enhance blood flow might be higher for patients with femur fracture than for those with tibia fracture because the femur has more abundant adjacent soft tissues and inherent vascularity supplied from the surroundings compared to those of the tibia. Our data indicate that the effect of CO_2_ therapy in enhancing blood flow is evident even in smokers. We consider that the blood flow in smokers who tend to have less vascularity than non-smokers can also be increased by the CO_2_ therapy. However, the small number of patients in the present study should be considered while interpreting the results. These issues will be the target of future large-sized clinical trials with more homogeneous populations.

This study has some limitations. The sample size was small and included a heterogeneous population of patients. We included patients with femur and tibia fractures and those with fresh fractures and nonunions in accordance with the nature of this study (an early-phase clinical trial). It was evident that CO_2_ therapy promoted an increase in blood flow in the fractured limbs of patients; however, it remains to be determined whether this increase has a direct positive effect on fracture repair. Currently, we do not possess radiological data to confirm the acceleration of fracture repair because this clinical trial was designed mainly to assess the safety. The true endpoint of studies investigating fracture repair is the acceleration of bony union, and accordingly, this would be a target for further studies. Moreover, the measurement of blood flow using a laser Doppler blood flow meter reflects superficial micro-circulation; however, in our previous study on healthy volunteers using phosphorus-31 magnetic resonance spectroscopy, we found that CO_2_ therapy affected the deep tissue, triceps surae muscle, via changes in intramuscular pH [26].

Nevertheless, we believe that the questions we sought to answer in this study, namely, whether CO_2_ therapy is safe and effective to increase blood flow in the fractured limbs of patients, have been satisfactorily addressed. Given that CO_2_ therapy increases blood flow, this type of therapy is expected to be beneficial for the treatment of open fractures, fractures in patients with ischemic disease or diabetes mellitus, fractures in smokers, and avascular nonunions. In addition to an increase in blood flow, local oxygenation via the Bohr effect [26] is also expected to contribute to tissue healing. Moreover, positive effects related not only to the healing of bone but also to that of soft tissue can be expected. Whether CO_2_ therapy accelerates fracture repair, improves union rate, and shortens the time to union are still unclear, which necessitates further study; however, we believe that CO_2_ therapy is a promising new clinically applicable tool that can be used to assist fracture repair.

## Conclusions

The topical cutaneous application of carbon dioxide via hydrogel has been shown to be clinically safe and to promote blood flow in fractured limbs in a small sample of patients.

## Data Availability

The datasets used and/or analyzed during the current study are available from the corresponding author upon reasonable request.

## Abbreviations

CO_2_: carbon dioxide
IM: intramedullary
